# Expanding the role of nurses in primary health care: the case of Brazil*

**DOI:** 10.1590/1518-8345.0000.3245

**Published:** 2019-12-05

**Authors:** Silvia Helena De Bortoli Cassiani, Fernando Antonio Menezes da Silva

**Affiliations:** 1 Pan American Health Organization, Washington, DC, USA.

**Figure:**
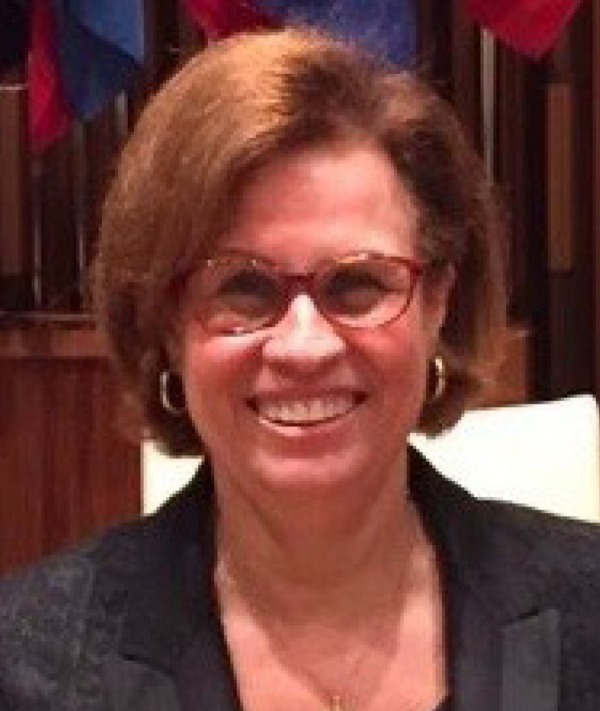

**Figure:**
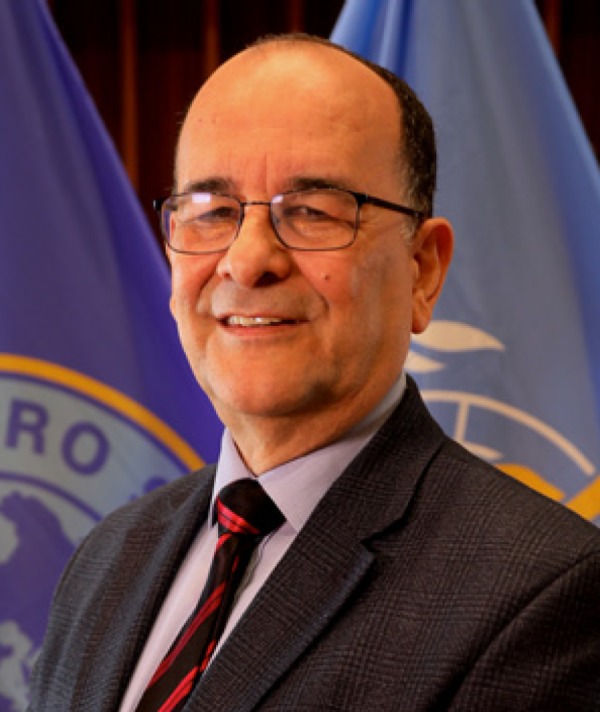

Brazil has 414,712 nurses^(^ 1 ^)^ and annually produces about 50,000 nurses in their nearly 1000 undergraduate nursing programs. It is the country with the largest number of nursing schools in the region of the Americas and graduate programs in nursing: master’s and doctorate, among Latin American Countries and the Caribbean. Of the total graduate programs in nursing (53) in this region, 72% (38) are in Brazil^(^ 2 ^)^. It is one of the only countries that has trained nurses in professional master’s degrees, which grew 156% in the period 2011-2020.

The Report of the Coordination for the Improvement of Higher Education Personnel (CAPES), a foundation affiliated with the Ministry of Education of Brazil, from 2017^(^ 3 ^)^, indicates that the professional master’s degree “is focused on the labor market and intends to respond to the needs for advanced and transformative professional training for a productive system and service delivery sector, both considering public and private organizations, seeking to respond to specific identified problems, in order to contribute to the socioeconomic and cultural development at the local, regional and national levels, in line with the guidelines and goals of the National Postgraduation Plan 2011-2020 and with the consolidation of the Unified Health System”. In 2017, there were 21 professional master’s degrees courses in nursing, in 2019, there are already 24, with potential for growth in the coming years**.

In 2018, the Pan American Health Organization/World Health Organization (PAHO/WHO) launched a call to expand the role of nurses in primary health care^(^ 4 ^)^ and presented the advanced practice nurse as a graduate professional who, integrated with the interprofessional team of primary health care services, contributes to the management of care of patients with mild acute diseases and chronic disorders, diagnosed according to the guidelines of protocols or clinical guidelines. The expanded scope of practice would be different from that performed by public health nurses due to the degree of autonomy in decision-making and the diagnosis and treatment of the patient’s disorders.

The expansion of the role of nurses in primary health care does not in any way intend to replace any other health professional; on the contrary, it intends to complement it, in addition to helping the population to have access to qualified health care professionals.

Advanced practice nurses are already regulated in Australia, Belgium, Canada, Finland, France, Ireland, Japan, Poland, the Czech Republic, the United Kingdom and the United States. In Spain, the Netherlands, the United Kingdom and Switzerland, political interest in expanding the role of nurses has risen due to the deficit of family doctors, changes in health systems and the creation of new models of health care^(^ 4 ^)^. Chile and Mexico have advanced in the process of implementation of advanced practice nurses.

In general, the most advanced functions or roles of nursing have been implemented in countries to improve access to care in regions with limited access to physicians, to maximize access to primary health care, and to enable intensive monitoring of patients with chronic diseases. In Iceland, an outpatient nursing service was implemented in which the role of nurses was expanded to meet the needs of patients with diabetes mellitus^(^ 4 ^)^.

Advanced practice nurses play a decisive role in primary care, have a high rate of user satisfaction and cost reduction. Scientific evidence demonstrates their impact on services and health costs. However, and paradoxically, the most economically developed countries with the highest number of physicians per population are those that have incorporated advanced practice nurses, not only in primary health care services, but also in hospitals.

The Organization for Economic Cooperation and Development (OECD) reports that many of its member countries have implemented reforms to expand the profile and practice of nurses. The roles of advanced practice nurses, according to this Organization, can be divided into three categories: 1. nurses acting in advanced roles as generalists to overcome the shortage of doctors or geographical barriers; 2. nurses acting primarily in the prevention and promotion of health and 3. nurses acting primarily in advanced roles as clinical specialists for the treatment and monitoring of clients, preferably with chronic conditions (e.g. diabetes or coronary artery disease)^(^ 5 ^)^.

The Nursing Now campaign, led by the English Parliament, the International Nursing Council and with the participation of the World Health Organization (WHO), launched in February of 2018, with its patron the Duchess of Cambridge, highlights the urgent need to elevate the profile of nurses and allow the professional to develop its full potential. One of the goals of the Campaign, which is expected to end in 2020, is investments in the nursing workforce in order to fully reach the potential of nursing practice to transform the model of health care.

The report the State of World’s Nursing which will be launched by WHO on World Health Day in 2020 will explore, through data, the situation of countries in implementing advanced functions for nurses. We can already predict that the region of Latin America and the Caribbean will have little to show.

At a time when countries, such as Brazil, are discussing the population’s access to qualified professionals, advanced practice nurses in primary health care can be a considerable alternative. Populations with difficulties due to geographical distances, socioeconomic barriers, or other factors related to access to health care services could benefit from the service of a well-prepared nurse trained at the graduate level, in accredited universities with advanced competencies and training in primary health care.

The employment of advanced practice nurses to fill gaps in health systems and services and the skill-mix of the health workforce offer the possibility to launch a new initiative in countries within the framework of universal access to health and universal health coverage.

The role of the advanced practice nurse, with autonomy, title recognition and supervision of a physician, even from a distance, can mean a new direction for healthcare in Latin America and the Caribbean. 

Brazil has all of the conditions to expand, recognize, value and broaden the role of nurses in primary health care. This will require a strong and integrated effort by all: government, professional associations, universities and other actors in raising awareness and discussion among professionals from the health team and the population in general. It could signify a new direction in health care practices within the country, like it was done in other countries over 50 years ago!

## Footnotes

** Personal communication from the Coordinator of the Nursing Area in CAPES, on August 22, 2019, received by electronic mail.
